# The contribution of walking to work to adult physical activity levels: a cross sectional study

**DOI:** 10.1186/1479-5868-11-37

**Published:** 2014-03-11

**Authors:** Suzanne Audrey, Sunita Procter, Ashley R Cooper

**Affiliations:** 1 School of Social and Community Medicine, University of Bristol, Canynge Hall, Whatley Road, Bristol BS8 2PS, UK; 2 Centre for Exercise Nutrition and Health Sciences, School for Policy Studies, University of Bristol, Bristol BS8 1TZ, UK; 3 National Institute for Health Research Biomedical Research Unit in Nutrition, Diet and Lifestyle, University Hospitals Bristol NHS Foundation Trust and the University of Bristol, Bristol, UK

**Keywords:** Physical activity measurement, Accelerometer, Walking, Adult physical activity guidelines

## Abstract

**Objective:**

To objectively examine the contribution to adult physical activity levels of walking to work.

**Methods:**

Employees (n = 103; 36.3 ± 11.7 years) at 17 workplaces in south-west England, who lived within 2 miles (3.2 km) of their workplace, wore Actigraph accelerometers for seven days during waking hours and carried GPS receivers during the commute to and from work. Physical activity volume (accelerometer counts per minute (cpm)) and intensity (minutes of moderate to vigorous physical activity (MVPA)) were computed overall and during the walk to work.

**Results:**

Total weekday physical activity was 45% higher in participants who walked to work compared to those travelling by car (524.6. ± 170.4 vs 364.6 ± 138.4 cpm) and MVPA almost 60% higher (78.1 ± 24.9 vs 49.8 ± 25.2 minutes per day). No differences were seen in weekend physical activity, and sedentary time did not differ between the groups. Combined accelerometer and GPS data showed that walking to work contributed 47.3% of total weekday MVPA.

**Conclusions:**

Walking to work was associated with overall higher levels of physical activity in young and middle-aged adults. These data provide preliminary evidence to underpin the need for interventions to increase active commuting, specifically walking, in adults.

## Introduction

There is compelling evidence that regular physical activity is effective in the prevention of chronic diseases (including cardiovascular disease, type 2 diabetes, some cancers, hypertension, obesity, depression and osteoporosis) and premature death, with the greatest improvements in health status seen when people who are least active become physically active [1,2]. In the United Kingdom (UK) it is currently recommended that adults should aim to undertake at least 150 minutes of moderate intensity physical activity in bouts of 10 minutes or more throughout the week [3,4] but many adults in the United Kingdom and other high-income countries do not achieve this [1,4-6]. Increasing physical activity levels, particularly among the most inactive, is an important aim of current public health policy in the UK [1,7-9].

### The benefits of active travel

One approach to increasing physical activity levels is to promote active travel i.e. walking and cycling. There is increasing evidence of the link between adult obesity levels and travel behaviour, one indicator of which is that countries with highest levels of active travel generally have the lowest obesity rates [10]. Experts in many World Health Organisation (WHO) countries agree that significant public health benefits can be realised through greater use of active transport modes [11]. For example, a systematic review of trials and cohort studies found modest but consistent support for the positive health effects of active travel, including a suggested positive effect on diabetes [12]. Other studies have shown a protective association between active travel and cardiovascular risk [13,14] and perceived health status [15]. Furthermore, cost benefit analysis for the UK Department for Transport suggests the ratio of benefits to costs were high [16]. The suggested benefits to employers of promoting active travel schemes include: increased productivity, a reduction in sick leave, improved public image as a result of lowering the workplace’s carbon footprint, and savings in providing car parking facilities [14,17-20].

### Walking as active travel

Walking has been described as near perfect exercise [21]. It is a popular, familiar, convenient and free form of exercise that can be incorporated into everyday life and sustained into older age. It is also a carbon neutral mode of transport that has declined in recent decades in parallel with the growth in car use [1]. Even walking at a moderate pace of three miles/hour (five km/hour) expends sufficient energy to meet the definition of moderate intensity physical activity [22]. Hence there are compelling reasons to encourage people to walk more, not only to improve their own health but also to address the problems of climate change [23-26].

In the UK, there are substantial opportunities to increase walking by replacing short journeys undertaken by car. For example, the 2011 National Travel Survey showed 22% of all car trips were shorter than two miles (3.2 km) in length, while 18% of trips of less than one mile were made by car [27]. An opportunity for working adults, especially those who live relatively close to their workplace, to accumulate the recommended moderate activity levels may be through the daily commute.

Although cycling is also an important mode of active transportation, walking may be perceived as a cheaper and safer option for those who are currently inactive: it requires no special equipment and is less likely to involve direct competition with motorised traffic for road space. In addition, for longer journeys, walking can more easily be combined with other transport modes such as buses and trains. In their study promoting active travel to work, Mutrie et al. (2002) [28] found the intervention group almost twice as likely to report an increase in walking during their journey to work as the control group at six months (odds ratio of 1.93, 95% confidence intervals 1.06 to 3.52) but there was no increase in cycling.

### Measuring active travel

Active travel has been associated with increased physical activity in studies using self report [29]. However, a systematic review comparing direct versus self-report measures for assessing physical activity in adults found self-report measures were both higher and lower than directly measured levels [30]. This questions the validity and reliability of self-report measures, and also undermines efforts to correct for self-report differences. However, very few studies have objectively measured the contribution of walking, particularly walking to work, to adult physical activity levels [31]. In the US, a cross-sectional study included 2,364 participants enrolled in the Coronary Artery Risk Development in Young Adults (CARDIA) study who worked outside the home during year 20 of the study (2005–2006) [32]. Associations were examined between walking or cycling to work and objective MVPA using accelerometers and active commuting was found to be positively associated with fitness in men and women, and inversely associated with BMI, obesity, triglyceride levels, blood pressure and insulin level in men. The authors concluded that active commuting should be investigated as a means of maintaining or improving health.

Objective measures of physical activity are more common in studies examining children’s commute to school. Studies investigating differences in physical activity between children who walk to school and those who travel by car have shown that children who walk to school have substantially higher physical activity than car travellers [33]. More recently, longitudinal studies have shown that a change of travel mode from passive (car/bus) to active (walking) is associated with an increase in overall daily physical activity, whilst physical activity declines if children adopt car travel instead of walking to school [34,35]. Spatial segmentation studies have confirmed the importance of walking to school to children’s overall physical activity, showing that approximately a third of daily MVPA is acquired in the school journey [36].

### The walk to work study

In the UK, public health guidance on workplace health promotion from the National Institute for Health and Clinical Excellence (NICE) has asserted that, although a range of schemes exist to encourage employees to walk or cycle to work, little is known about their impact and the measures of physical activity used are often based on self-report [37]. In this context, the Walk to Work feasibility study [38] was developed in the south-west of England using objective measurements of physical activity. The main study is examining the feasibility of implementing and evaluating an intervention through which Walk to Work promoters are recruited and trained to encourage fellow employees, who do not currently walk or cycle to work, to increase the amount of walking they undertake during the daily commute. This paper focuses on baseline data to examine the association between travel mode to work and objectively measured physical activity in adults.

## Methods

### Recruitment

Workplaces were contacted by email through the local Chambers of Commerce and by post through a publicly available list of employers in the area. Each workplace was sent an information sheet about the Walk to Work study and asked to return a form to indicate expressions of interest. Following additional information about what the study entailed, a total of 17 workplaces were recruited to the study: eight small (≤ 50 employees), five medium (51–250 employees) and four large (>250 employees).

Eligible employees were adults in full or part-time employment who lived within two miles (just over three kilometres) of their workplace and were capable of walking to work regardless of their current mode of transport for commuting. Participating workplaces were asked to identify eligible employees by matching postcodes of workplace and home address and calculating distance using an online calculator (http://Walkit.com). It was a requirement of the research ethics committee that this was done by workplaces and not the research team. Eligible employees were then given an information sheet and a letter of invitation to take part in the study. Written informed consent was obtained from each participant before data collection commenced.

The study was given ethical approval by the University of Bristol Faculty of Medicine and Dentistry Research Ethics Committee.

### Measurements

Baseline data were collected between May and July 2012. Participants were asked to complete questionnaires giving basic personal data including age, sex, ethnicity, household income, educational attainment and job characteristics (Table 1). Physical activity was measured objectively using accelerometers (Actigraph GT3X+; ActiGraph LLC, FL, USA) worn on a belt around the waist during waking hours for seven days and removed for swimming and bathing. Accelerometers were set to record data at 30 Hz. Participants also wore a personal Global Position System (GPS) receiver (QStarz BT1000XT) on the same belt during their commute to allow the journey to be spatially described. GPS data were recorded at 10-second intervals and the “assisted GPS” mode was used to enhance the precision of the GPS location data. Participants also recorded details (mode and duration) of each journey to and from work in a travel diary.

**Table 1:** Demographic characteristics of participants by mode of travel to work

	**Walk (n = 70)**	**Car (n = 33)**	**All (n = 103)**
Age (years)	36.8 ± 12.0	35.4 ± 11.1	36.3 ± 11.7
**Sex (%)**			
Male	40.0	48.5	42.7
Female	60.0	51.5	57.3
**Ethnicity (%)**			
White	92.8	96.9	95.1
**Household income (%)**			
<£10,000	4.4	0	3.0
£10,001–£20,000	14.7	6.5	12.1
£20,001–£30,000	20.6	16.1	19.2
£30,001–£40,000	13.2	12.9	13.1
£40,001–£50,000	16.2	6.5	13.1
>£50,000	25.0	41.9	30.3
Not disclosed	5.9	16.1	9.1
**Education (%)**			
No formal education	1.4	3.1	2.0
GCSE grades A-C, GCE “O” level, CSE grade 1, NVQ2 or equivalent	7.2	12.5	8.9
BETC (national), BEC (national) TEC (national), ONC, OND or equivalent	5.8	3.1	5.0
GCE “A” level. NVQ3, Scottish higher or equivalent	11.6	15.6	12.9
BETC (higher, TEC (higher), HNC, HND or equivalent	1.4	3.1	2.0
Degree, NVQ4 or equivalent	49.3	34.4	44.6
PhD, Masters, NVQ level 5 or equivalent	23.2	28.1	24.8
**Occupation (%)**			
Sedentary	80.9	78.8	80.2
Standing	14.7	18.2	15.8
Manual	2.9	3.0	3.0
Heavy manual	1.5	0	1.0
**Employment pattern (%)**			
Full time	90.8	80.6	87.5
Part time	9.2	19.4	12.5

Participants were given brief instructions in the workplace about how to use the equipment by a member of the research team when the data collection equipment was distributed. Brief written instructions were also supplied, with the contact details of the research team in case of any problems or queries. All participants who returned questionnaires, travel diaries, accelerometers and/or GPS data were given a £10 gift voucher (approximately €12 or $16) to acknowledge their contribution to the study.

### Data reduction

Raw accelerometer data were downloaded using Actilife 6 software (ActiGraph LLC) and reintegrated to ten-second epochs for analysis and matching with GPS data. Reintegrated accelerometer data were processed using Kinesoft (v3.3.62; KineSoft, Saskatchewan, Canada) data reduction software to generate outcome variables. Continuous periods of 20 minutes or more of zero values were considered to be “non-wear” time and removed. Outcome variables were total physical activity volume (mean daily accelerometer counts per minute (cpm)), moderate to vigorous physical activity (MVPA) and sedentary time, defined using validated thresholds (MVPA >1952 cpm; sedentary <100 cpm) [39].

Participants were required to provide at least 600 minutes of accelerometer data for a single day to be considered valid, and all valid days were included in analyses. Accelerometer and GPS data were combined (accGPS) based upon the timestamp of the Actigraph data. For measurement of the journeys to and from work, the participant’s workplace and home were geocoded using the full postcode, and imported into a Geographical Information System (ArcMap v10). The merged accGPS files were then imported into ArcMap and journeys to and from work visually identified and segmented from other accGPS data using the “identify” tool. Journeys were identified as a continuous (or near-continuous) sequence of GPS locations between the participant’s home and workplace, and thus included trips to other destinations (e.g. supermarkets) if taken as part of the journey to or from work.

### Data analysis

Travel diaries were used to categorise participants by their “usual” mode of travel to work over the measurement week. Only days where participants reported using the same mode of transport both to and from work were included in analyses, and participants were categorised according to the most commonly used mode of transportation. Of the 147 participants who lived within two miles (3.2 km) of their workplace, 23 did not provide diary data, with the remainder providing 511 weekdays of travel data comprising: walk (244 days), car (102 days), cycle (72 days) and other/mixed (93 days). Participants were categorised as “usual walkers” (n = 68), “usual drivers” (n = 29), “usual cyclists” (n = 18) or “mixed/other” (n = 9). Participants who cycled to work were excluded from further analyses due to the inability of waist worn accelerometers to accurately record physical activity during cycling, as were data from participants using other/mixed modes of travel. Where a travel diary was not completed, usual travel mode was determined from the baseline behavioural questionnaire where possible (walk n = 6; car n = 4). The sample for analysis comprised 74 participants who usually walked to work, and 33 who usually commuted by car. Four of these participants did not provide any valid accelerometer data, and were excluded from analyses.

Analyses were confined to data recorded between 6.00 am and midnight. Mean (SD) values were computed for continuous variables and normal distribution confirmed. Differences in physical activity between travel modes (walk/car) were analysed by one-way ANOVA. Paired samples t-tests were used to compare weekday and weekend values for total physical activity (cpm), MVPA and time spent sedentary, and to investigate differences in the volume of MVPA accumulated between overall accelerometer data and spatially segmented trips. Linear regression was used to explore the association between travel mode (walk/car) and total weekday physical activity (cpm) and MVPA (minutes per day). Models were adjusted for possible confounders (age, sex, education (educated to degree level or not), income (salary below or above £30,000 per year (representing below and above mean UK household income)), work status (full/part time), occupational activity (sedentary/active)), and accelerometer wear time. Finally, one way ANOVA was used to compare total physical activity on all walking days with all car travel days.

## Results

The characteristics of workplaces recruited to the study are outlined in Table 2. There was a mix of small (n = 8, 47%), medium (n = 5, 29%) and large (n = 4, 24%) workplaces in predominantly urban areas although some suburban workplaces were included (n = 4, 24%). Just over half were office-based businesses (n = 9, 53%) but manufacturing, transport, catering and educational establishments were also included.

**Table 2:** Participating workplaces by size, type of business, location

**Workplace**	**Size**^**1**^	**Type of Business**^**2**^	**Location**
11	Small	Professional, scientific & technical	City centre
12	Small	Manufacturing	City centre
13	Small	Professional, scientific & technical	City centre
14	Small	Professional, scientific & technical	City centre
15	Small	Professional, scientific & technical	City centre
20	Small	Professional, scientific & technical	Suburban
21	Small	Transportation	Suburban
22	Small	Professional, scientific & technical	City centre
17	Medium	Education	Suburban
18	Medium	Professional, scientific & technical	City centre
19	Medium	Accommodation & food services	City centre
23	Medium	Manufacturing	City centre
24	Medium	Education	City centre
25	Large	Public administration	City centre
26	Large	Manufacturing	City centre
27	Large	Financial & insurance activities	City centre
28	Large	Manufacturing	Suburban

^1^Size: Small <50; Medium 51-250; Large >250.

^2^Office of National Statistics, UK Standard Industrial Classification of Economic activities 2007 (SIC 2007) [40].

The final sample comprised 103 adults (mean age 36.3 ± 11.7 yrs; 57.3% female) of whom 70 (50.3%) were categorised as walkers and 33 (22.4%) as car users. Participants were predominantly white, well educated and employed in sedentary (desk-based) occupations (Table 1).

There were no statistically significant differences in any demographic characteristic between the groups. Participants included in the final sample who completed the travel diary (n = 94) recorded 236 return journeys to work by foot, 95 by car, eight by bicycle and 56 using mixed modes. There was no record for 77 journeys. Mean self-reported journey time to work (single trip) was 19.7 ± 8.3 minutes by foot and 10.7 ± 7.6 minutes by car.

Participants wore the accelerometer for a mean of 721.6 ± 155.2 minutes each day, with no difference between travel modes or sexes. Physical activity did not differ between males and females (497.9 ± 206.0 versus 455.0 ± 150.9 cpm respectively; p = 0.224) and thus the sexes were analysed together. When analysed by main travel mode (Table 3), participants who walked to work had higher levels of overall weekday physical activity compared with those who travelled by car and also recorded more minutes of MVPA, but there was little difference in sedentary time.

**Table 3:** Weekday and weekend physical activity by usual travel mode to work on weekdays (mean ± standard deviation)

	**All**	**Walk**	**Car**	**p**
**Weekday**	**(n = 103)**	**(n = 70)**	**(n = 33)**	
Overall daily physical activity (accelerometer counts per minute (cpm))	473.3 ± 176.9	524.6 ± 170.4	364.6 ± 138.4	<0.001
Moderate to vigorous physical activity (MVPA; minutes/day)	69.0 ± 28.2	78.1 ± 24.9	49.8 ± 25.2	<0.001
Sedentary time (minutes/day)	586.8 ± 71.9	581.0 ± 76.3	599.2 ± 60.5	0.231
**Weekend**	**(n = 68)**	**(n = 46)**	**(n = 22)**	
Accelerometer counts per minute	413.2 ± 195.9	426.7 ± 211.7	385.1 ± 158.6	0.417
Moderate to vigorous physical activity (MVPA; minutes/day)	53.1 ± 30.2	54.5 ± 31.6	50.2 ± 27.7	0.590
Sedentary time (minutes/day)	517.4 ± 106.0	521.4 ± 113.6	509.0 ± 90.0	0.657

At the weekend, total physical activity and MVPA were substantially lower in those who walked to work during the week compared to weekday values, but the physical activity of car users was essentially unchanged. There was no difference in weekend physical activity between the two travel groups. In linear regression models walking to work was associated with higher overall weekday physical activity and MVPA, contributing to 19 minutes of additional MVPA each day in adjusted models, but not sedentary time (Table 4).

**Table 4:** Linear regression analysis of total weekday physical activity, MVPA and sedentary time with travel mode

	**Total physical activity**		**MVPA**		**Sedentary time**	
	**β (95% CI)**	**p**	**β (95% CI)**	**p**	**β (95% CI)**	**p**
Sex (male (reference)/female)	−25.4 (-104.3, 53.6)	0.524	1.8 (-10.1, 13.7)	0.766	0.3 (-29.6, 30.2)	0.984
Age (years)	−3.0 (-6.3, 0.3)	0.077	−0.3 (-0.8, 0.2)	0.198	1.0 (-0.3, 2.2)	0.119
Education (no university degree (reference)/degree)	43.8 (-41.8, 129.4)	0.311	8.6 (-4.3, 21.5)	0.188	3.0 (-29.4, 35.4)	0.854
Income (≤£30,000 per annum (reference)/>£30,000)	−47.2 (-127.2, 32.7)	0.243	−6.7 (-18.8, 5.3)	0.268	44.3 (14.0, 74.6)	0.005
Occupational activity (sedentary (reference)/non-sedentary)	−41.5 (-138.0, 55.0)	0.394	−15.6 (-30.1, -1.1)	0.036	−39.6 (-76.2, -3.1)	0.034
Work status (part time (reference)/full time)	53.4 (-66.0, 172.7)	0.376	15.1 (-2.9, 33.0)	0.099	10.5 (-34.7, 55.7)	0.646
Accelerometer wear time (minutes per day)	0.13 (-0.15, 0.40)	0.364	0.06 (0.02, 0.10)	0.008	0.17 (0.07, 0.28)	0.002
Travel mode (car (reference)/walk)	127.3 (43.9, 210.8)	0.003	19.0 (6.4, 31.6)	0.004	−15.6 (-47.2, 16.0)	0.327

Regression results are presented as unstandardised coefficients (95% CI). Total weekday physical activity is accelerometer counts per minute (cpm), MVPA and Sedentary time are minutes.

**Figure 1:**
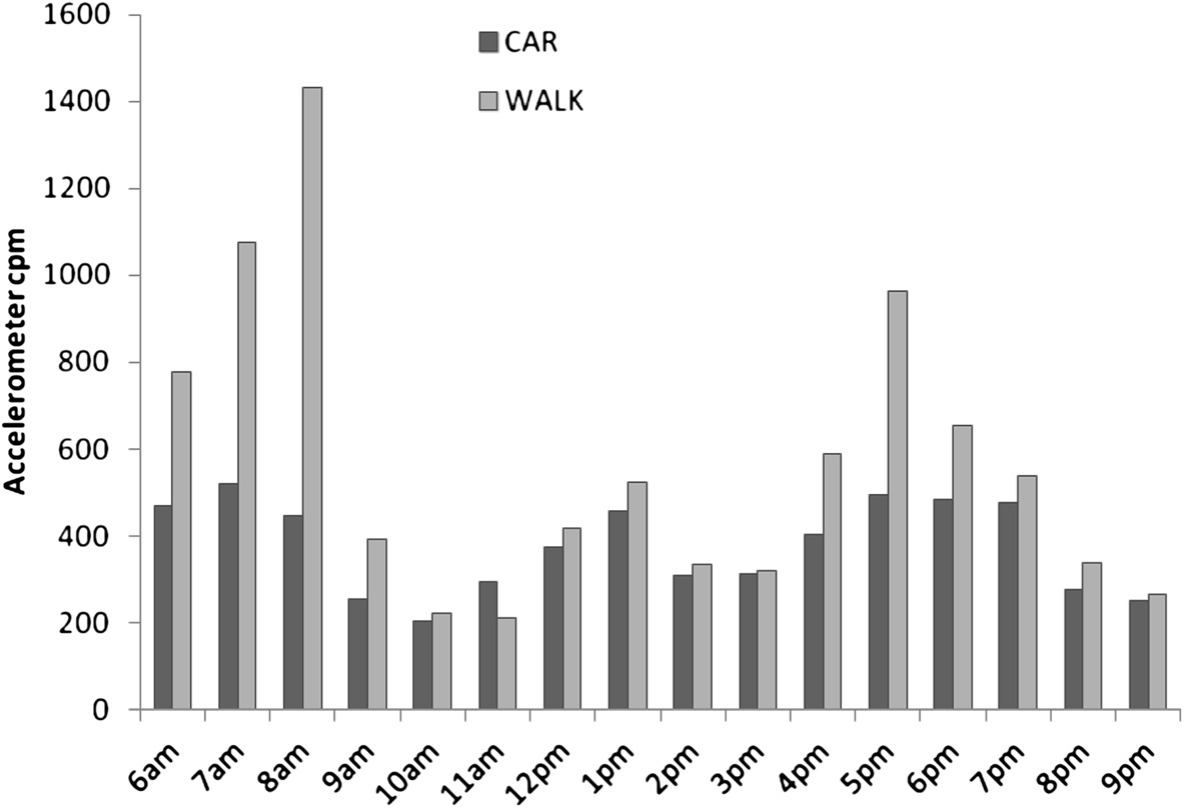
Mean hourly physical activity by mode of travel to/from work on weekdays.

Mean hourly physical activity was plotted to identify when differences in physical activity occurred during the day. Figure 1 shows that the main differences between car users and walkers occurred in the morning and late afternoon, potentially when commuting to or from work. There was no difference in physical activity between the travel groups during the main working hours (9 am to 4 pm) (walk: 347.2 ± 187.4 cpm vs car: 318.7 ± 194.4 cpm; p = 0.480). To further explore the level of physical activity associated with walking to work, all days where the journey was conducted by foot were compared with all days where the journey was by car. Average physical activity on walking days was substantially higher than car days (583.1 ± 182.4 vs 319.7 ± 148.5 cpm; p < 0.001), with values similar to those in the analysis by individual shown in Table 3.

To explore the contribution of walking to work to total physical activity, accGPS traces recorded between 6.00 am and 10.00 am, and between 4.00 pm and 8.00 pm, were examined. Of the 74 participants who walked to work, 58 recorded GPS data for at least one journey. Overall, 321 journeys (182 to work, and 139 home from work) were recorded. Participants spent almost 22 minutes walking to work and 29 minutes walking home (21.9 ± 7.8 vs 28.6 ± 18.5 minutes), reflecting longer routes taken to home in order to visit shops. These visits were considered to be part of the journey. Average physical activity was high during both journeys (to/from work: 4260.7 ± 943.5 vs 3806.3 ± 915.8 cpm), though less on the journey home due to visits to shops. However, the minutes of MVPA on both the journey to and from work were similar (19.8 ± 7.1 vs 21.0 ± 8.9 minutes of MVPA) since time spent in shops was not MVPA. Comparison of total MVPA (6.00 am to midnight) with MVPA recorded during the journey for the 58 participants providing any GPS data showed that the walk to and from work contributed 47.3% of participants total daily MVPA (38.0 of 80.3 minutes).

## Discussion

This study explored the potential contribution of walking to work to daily physical activity in adults. In particular, we compared the baseline physical activity data from participants in a larger study to examine differences between walkers and car users. We found that activity levels were 44% higher in participants who walked to work than those travelling by car, and accumulated 57% more MVPA. No differences were seen in physical activity during working hours or at weekends between walkers and car users. Hourly activity patterning showed that the difference between walkers and car users in weekday physical activity predominantly occurred during commuting hours, and spatial segmentation showed that the journey to and from work was responsible for the majority of the difference in weekday physical activity between those who walked to work and those who travelled by car.

An important strength of this study is the combined use of accelerometry and GPS to measure the journey to work. Whilst accelerometers are commonly used to measure physical activity and can provide highly time resolved data, they are unable to record the context of physical activity. Consequently estimates of physical activity based upon, for example, hourly mean physical activity (as illustrated in Figure 1) may also include other physical activities taking place around the journey (for example walking a dog before walking to work). Combining accelerometer data with positional data from GPS receivers allowed both the level and location of physical activity to be described, and permitted identification of activity levels specifically during journeys or in particular places. These data showed that where participants took longer routes home (for example, visiting shops en route) this did not necessarily contribute to daily MVPA. Thus judging the contribution of journeys to MVPA based upon duration may be prone to error.

The study took place in a range of small, medium and large workplaces that engaged in different types of activities. As such, the study aimed to address a gap in the current research literature: the range of settings covered has been very limited and, in particular, evidence is lacking about small and medium-sized enterprises [37]. However, there are also a number of limitations to this study. The data were collected as part of a feasibility study for which we recruited a relatively small sample of predominantly well-educated younger adults, limiting the generalisability of the findings. Larger, more representative studies using objective methods are needed. The results also show high levels of MVPA, which may partly be a reflection of the accelerometer threshold used (although the threshold used is commonly applied in many studies) but also of the demographic profile of the participants. Nevertheless, the potential contribution to physical activity levels of walking the daily commute is clearly illustrated.

To our knowledge, ours is the first study in adults to use accelerometers and GPS spatial segmentation to quantify the contribution of walking to work, and our findings are consistent with those in children, demonstrating the substantial contribution that walking to work can make to daily physical activity. These data provide persuasive evidence to underpin interventions to increase active commuting in adults.

Public Health England recently announced several ‘high-level enduring priorities’ guiding their work, two of which are relevant to this study: helping people to live longer and more healthy lives by reducing preventable deaths, and; improving health in the workplace by encouraging employers to support their staff, and those moving into and out of the workforce, to lead healthier lives [41]. Similar aims are shared by governments and health practitioners throughout the world. The data presented here suggest that encouraging employees to walk to work has the potential to make a contribution to addressing these priorities.

## Competing interests

The authors declare that they have no competing interests.

## Authors’ contributions

SA conceived and designed the study and wrote the first draft of the paper. AC analysed the accelerometer and GPS data and helped to draft the manuscript. SP coordinated the data collection and helped to draft the manuscript. All authors read and approved the final document.
